# CBCT-Based Epidemiological Study of Root and Root Canal Anatomy in Mandibular Second Molars in an Italian Clinical Cohort

**DOI:** 10.3390/jcm15103688

**Published:** 2026-05-11

**Authors:** Katia Greco, Riccardo Federico Visconti, Gaetano Paolone, Maria Teresa Sberna, Enrico Felice Gherlone, Giuseppe Cantatore

**Affiliations:** IRCCS San Raffaele Scientific Institute, Vita-Salute San Raffaele University, Dental School, Department of Endodontics, 20132 Milan, Italy; katiagreco@libero.it (K.G.); riccardo.visconti@hotmail.it (R.F.V.); paolone.gaetano@hsr.it (G.P.); sberna.mariateresa@hsr.it (M.T.S.); gherlone.enrico@hsr.it (E.F.G.)

**Keywords:** cone-beam computed tomography (CBCT), mandibular second molars, root canal configuration, C-shaped canals, Vertucci classification, Fan classification, bilateral symmetry

## Abstract

**Background**: Mandibular second molars show notable variability in root canal structures and C-shaped morphology, with possible differences among populations. **Methods**: This retrospective cross-sectional CBCT study included 500 patients attending the Department of Dentistry at IRCCS Ospedale San Raffaele (Milan, Italy) with bilateral mandibular second molars and was reported according to STROBE guidelines. CBCT scans (Hyperion X5; voxel size 0.125 mm) were assessed by two endodontists using standardized criteria. Root-based canal configurations were classified according to Vertucci in cases with complete bilateral coding of homologous mesial and distal roots; C-shaped morphology was classified using Fan’s system and analyzed separately because Vertucci coding is not applicable to C-shaped systems. Categorical variables were analyzed using χ^2^ or Fisher’s exact test, continuous variables with parametric or non-parametric tests, and right–left comparisons with paired-sample tests (*p* < 0.05). **Results**: Complete bilateral Vertucci coding was feasible in 494/500 patients (98.8%), yielding 988 mesial and 988 distal roots for analysis. C-shaped canal configuration was detected in 1.2% of patients (6/500; 95% CI 0.44–2.59%); females showed a higher proportion than males (2.0% vs. 0.4%), with no evidence of a sex association (Fisher’s exact test, *p* = 0.216). Fan subtype annotation was available for 5/6 patients and 7 teeth; C1, C3, and C4 patterns were observed. In the Vertucci dataset, mesial roots most frequently exhibited Types II (52.0%) and IV (26.5%), whereas distal roots were predominantly Type I (62.4%), followed by Type III (29.8%). Contralateral symmetry was observed in 27.3% of mesial roots (135/494; 95% CI 23.4–31.5%) and 59.1% of distal roots (292/494; 95% CI 54.6–63.5%). Mean pulp chamber roof-to-floor distance was 2.623 ± 0.263 mm on the right and 2.567 ± 0.343 mm on the left (paired *p* < 0.001; mean difference 0.056 mm; 95% CI 0.023–0.089 mm). **Conclusions**: In this cohort, C-shaped morphology was rare, and no evidence of a sex association was found, although the small number of cases limits statistical power. Mesial roots showed more variability than distal roots, and contralateral symmetry was moderate and greater for distal roots than for mesial roots, supporting contralateral anatomy as a helpful—rather than predictive—clinical reference.

## 1. Introduction

The success of endodontic treatment relies heavily on a thorough understanding of root anatomy and its variations, which is crucial for effective cleaning and three-dimensional obturation of the root canal system. Numerous studies have indicated that the complexity of the root canal system is linked to increased treatment difficulty and can affect long-term clinical outcomes [1,2]. In this context, mandibular molars—especially mandibular second molars—are of clinical interest due to their significant variability in root number and canal configuration [3].

The introduction of three-dimensional imaging has significantly transformed the study of endodontic anatomy. Conventional two-dimensional radiography, while widely used in clinical practice, has inherent limitations, including the superimposition of anatomical structures and the two-dimensional representation of a complex three-dimensional system [4,5]. Cone-beam computed tomography (CBCT), on the other hand, allows for three-dimensional assessment of dental and periradicular structures, making it easier to identify variations in the root canal system. Therefore, the guidelines of the European Society of Endodontology recommend the selective use of CBCT in endodontics, especially when complex anatomy is suspected and traditional imaging is inadequate [6].

**Figure 1:**
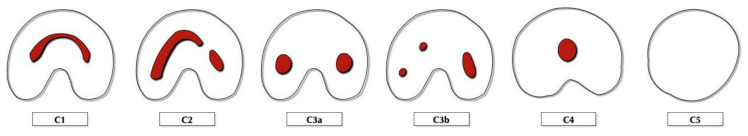
Schematic representation of C-shaped configurations (C1–C5) according to the classification by Fan et al., as observed on axial CBCT sections.

Among the most complex and clinically important anatomical variants of mandibular second molars, the C-shaped configuration stands out. This morphology was first described by Cooke and Cox in 1979 [7] and features partial or complete root fusion with a root canal system that, on cross-sections, appears as a continuous or broken “C” shape. Clinically, C-shaped canals are often linked to complicated canal systems that include lateral or accessory canals, anastomoses, and isthmuses, making shaping, cleaning, and filling especially difficult. A recent review focusing on mandibular second molars has further highlighted the significant morphological variability of both C-shaped and non-C-shaped root canal systems in this tooth group [8]. Several classification systems have been developed to describe C-shaped anatomies. Among them, the classification introduced by Fan et al. in 2004 is widely accepted and frequently used in CBCT-based epidemiological studies because it offers a standardized and reproducible way to describe configurations along the root length [9]. According to this system, C-shaped configurations are grouped into five major categories based on axial shape: C1 (continuous C-shaped canal), C2 (discontinuous or semicolon-shaped), C3 (divided into two subcategories, C3a with two separate canals and C3b with three separate canals), C4 (a single round or oval canal), and C5 (no detectable canal lumen, usually at the apex) (Figure 1).

CBCT-based epidemiological studies have shown significant ethnic differences in the prevalence of C-shaped configurations in mandibular second molars. Asian populations have a much higher prevalence, as reported in studies of Chinese and Korean populations [10,11], while European groups display lower rates and greater variability, as documented in studies from Portugal and Belgium [12,13]. A 2025 systematic review and meta-analysis further confirmed that the mandibular second molar has the highest pooled prevalence of C-shaped canal morphology among premolars and molars assessed by CBCT, with higher pooled values in women and in Asian populations, and no significant overall right–left difference [14]. Other recent large-scale analyses and systematic reviews have highlighted the importance of large sample sizes and standardized methods to improve comparability across populations and have confirmed extensive geographic and methodological variation in the distribution of C-shaped morphologies. [15,16]. However, despite Italy’s size and regional diversity, epidemiological data on the endodontic anatomy of mandibular second molars remain limited and relatively underrepresented in the global literature.

**Figure 2:**
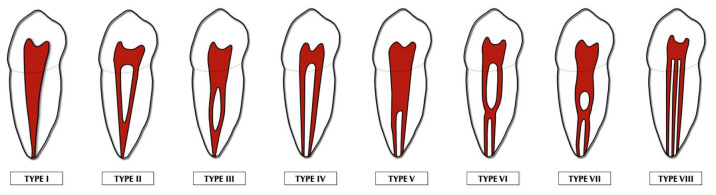
Schematic representation of the Vertucci root canal configuration classification (Types I–VIII). Each panel shows the canonical pattern of canal splitting and merging from the pulp chamber to the apical foramen.

This epidemiological study aimed to analyze the root and root canal anatomy of mandibular second molars in an Italian hospital-based clinical cohort using CBCT. The specific objectives were: (i) to classify canal configurations following Vertucci (Figure 2); (ii) to assess the prevalence and distribution of C-shaped morphologies based on Fan’s classification; (iii) to investigate potential differences by sex and laterality (right/left); and (iv) to examine bilateral symmetry and the distance from pulp chamber roof to floor.

The formal null hypotheses proposed that (i) no sex-related differences would be observed in the distribution of Vertucci canal configurations within this cohort, and (ii) no sex-related differences would be observed in the prevalence of C-shaped morphology across the entire cohort. Additionally, right–left patterns and contralateral symmetry were analyzed descriptively within the cohort.

## 2. Materials and Methods

### 2.1. Study Design and Ethical Considerations

This cross-sectional observational study was reported following the STROBE guidelines for observational studies [17]. The study was designed as a retrospective analysis of CBCT images previously obtained for diagnostic purposes in routine clinical practice. An a priori sample size calculation was conducted using G*Power version 3.1 [18] for the primary categorical comparisons of Vertucci canal configuration distributions. Assuming a chi-square test for contingency tables, a significance level of 0.05, a power of 0.80, 6 degrees of freedom, and a small-to-moderate effect size (Cohen’s w = 0.17), the minimum required total sample size was 472 subjects. The target sample was therefore rounded to 500 patients to ensure balanced sex representation and to maintain sufficient numbers for analysis after exclusions. All datasets were anonymized before analysis. Since this study involved secondary analysis of fully anonymized radiological images and did not include identifiable information or any additional patient procedures, formal ethics committee approval was not required per institutional policy and applicable regulations. Written informed consent for CBCT acquisition and personal data processing was obtained at the time of imaging; no additional consent was necessary for this anonymized retrospective analysis.

### 2.2. Study Population Selection

CBCT examinations were selected from a database of over 2000 scans acquired between January 2020 and December 2023 at the Dental Radiology Unit, Vita-Salute San Raffaele University. The scans involved patients attending the Department of Dentistry at IRCCS Ospedale San Raffaele, for surgical, periodontal, and endodontic diagnostic purposes. The final sample consisted of 500 patients with a balanced sex distribution (250 males and 250 females). Ages ranged from 19 to 71 years (overall mean 40.8 ± 10.0 years), with males aged 19–71 years (mean 41.3 ± 10.2 years) and females aged 22–61 years (mean 40.3 ± 9.7 years). Patients reported geographic origins from various regions of Italy, including Northern, Central, and Southern Italy, as well as the Islands.

### 2.3. Inclusion and Exclusion Criteria

Inclusion criteria included: bilateral mandibular second molars; complete root formation; no prior endodontic treatment; no extensive restorations; high-quality CBCT images without significant artifacts; and complete clinical and demographic information. Exclusion criteria involved teeth with extensive restorations, deep caries, developmental anomalies, immature roots, root resorption, visible periapical or periodontal lesions, and full-coverage crowns, as well as CBCT scans that were poor in quality or had significant artifacts. Strict criteria were used to analyze molars with unaltered root anatomy.

### 2.4. Unit of Analysis and Denominators

The primary sampling unit was the patient. Root morphology and the prevalence of C-shaped canal configurations were reported at the patient level. Root morphology for the right and left mandibular second molars was determined from serial axial CBCT sections and used to categorize patients based on bilateral anatomical eligibility for root-based analyses (i.e., the presence of distinct mesial and distal roots on both sides enabling Vertucci coding) versus non-eligible morphologies (e.g., fused or C-shaped systems). To prevent heterogeneous denominators and correlated observations, analyses of Vertucci canal configuration distributions and bilateral symmetry were limited to the Vertucci analysis dataset (*n* = 494), defined as cases with complete bilateral Vertucci coding for homologous mesial and distal roots. The six patients with C-shaped anatomy (6/500) were analyzed separately because the root-based Vertucci scheme does not apply to C-shaped systems. This homogeneous Vertucci subgroup produced 988 mesial roots and 988 distal roots, corresponding to right and left homologous roots (494 patients × 2 sides), for root-level analyses.

### 2.5. Classification of Root and Canal Morphology

Root number and morphology were recorded for each mandibular second molar on serial axial CBCT sections, supported by coronal and sagittal reconstructions. For root-based canal configuration analyses, Vertucci classification was applied only in the predefined Vertucci analysis dataset (*n* = 494), defined by complete bilateral Vertucci coding of homologous mesial and distal roots (right and left sides).

Within this dataset, canal configurations were classified separately for mesial and distal roots on both sides according to Vertucci by examining serial axial slices from the canal orifice level to the apical foramen. When a configuration changed along the root length, a single Vertucci type was assigned using a predefined rule: the most complex configuration observed at any level was retained, where “complexity” was operationally defined in advance as (i) the configuration displaying the highest number of distinct canals at any level and, when the maximum canal number was the same, (ii) the configuration showing the greatest number of splitting or merging events along the root trajectory. This rule was applied consistently by both examiners during calibration.

C-shaped canal morphology was assessed on axial sections along the entire length of the root. Teeth were classified as C-shaped if axial sections showed a continuous or discontinuous C-shaped pattern according to Fan’s definition and classification system. Teeth meeting the C-shaped criteria were classified using Fan’s system by examining serial axial sections and recording the observed subtypes along the root. Since Vertucci coding does not apply to C-shaped systems, C-shaped cases were analyzed separately at the patient level (prevalence) and, when available, at the tooth level (Fan subtypes).

### 2.6. CBCT Acquisition and Image Analysis

All CBCT scans were obtained using the Hyperion X5 3D radiography system (MyRay, Imola, Italy) with standardized parameters (90 kV, 10 mA, voxel size 0.125 mm, field of view 8 × 8 cm). Images were analyzed through multiplanar reconstructions (axial, coronal, and sagittal views). When necessary, datasets were reoriented to optimize visualization of the tooth’s long axis and to ensure consistent assessment of canal morphology along the root. Under standardized acquisition conditions, CBCT is deemed suitable for identifying major root canal configurations in epidemiological studies of endodontic anatomy [19].

### 2.7. Examiner Calibration and Measurements

Before the formal evaluation, a calibration phase was conducted on a set of CBCT examinations not included in the final dataset. Two experienced endodontists with over 10 years of clinical experience independently assessed the calibration scans using MyRay iRYS software version 6.4, with calibrated measurement tools previously validated in the literature [20]. The examiners were trained to (i) review serial axial sections along the entire root length, (ii) apply standardized criteria for identifying canals and root morphology, and (iii) follow the predefined rule for assigning a single Vertucci type when morphology changed along the root. Disagreements were resolved through joint re-evaluation until consensus was achieved, and the consensus code was used in the final dataset.

The variables recorded included: (a) root number and morphology (at the patient level); (b) Vertucci canal configuration for mesial and distal roots (Vertucci dataset only); (c) presence of C-shaped canal morphology and Fan subtype(s) (C-shaped cases only); and (d) pulp chamber roof-to-floor distance. For pulp chamber measurements, the linear distance was measured using the software ruler tool in the plane that provided the clearest identification of the roof and floor landmarks. Each measurement was repeated three times, and the average value was used for statistical analysis.

### 2.8. Statistical Analysis

Statistical analysis was conducted using IBM SPSS Statistics version 28.0 (IBM Corp., Armonk, NY, USA). The patient served as the primary sampling unit. Patient-level analyses were performed to report root morphology and the prevalence of C-shaped canal configuration (*n* = 500). Prevalence estimates were expressed as proportions and, when appropriate, accompanied by exact 95% confidence intervals (Clopper–Pearson).

Normality of continuous variables was assessed using the Shapiro–Wilk test [21]. Continuous variables were compared between independent groups using Student’s *t*-test (for normally distributed data) or the Mann–Whitney U test (for non-normally distributed data). Right–left comparisons of continuous measurements were evaluated with paired-sample tests (paired *t*-test for normally distributed paired differences; Wilcoxon signed-rank test otherwise). All tests were two-tailed, and statistical significance was defined as *p* < 0.05.

Because right and left homologous roots from the same patient are correlated, inferential analyses of Vertucci configuration by sex were performed separately for the right and left sides, ensuring that each test used independent observations (one root per patient per side). For each root type (mesial and distal), overall Vertucci distributions combining right and left sides (*n* = 988) were treated as descriptive summaries.

Vertucci configuration distributions and contralateral symmetry were analyzed within the predefined Vertucci analysis dataset (*n* = 494), yielding 988 mesial and 988 distal roots. Contralateral symmetry was defined at the patient level as an identical Vertucci configuration in contralateral homologous roots (right vs. left) and was reported as proportions for mesial and distal roots.

For categorical comparisons, Pearson’s χ^2^ test was used when expected cell counts were adequate; Fisher’s exact test was used when expected counts were less than 5. Missing data were handled by analysis-specific exclusion (complete-case analysis): for example, C-shaped laterality or Fan subtype was analyzed only in cases where that information was available, while prevalence analyses used the full patient denominator. Analyses involving C-shaped morphology were not used to determine sample size because this phenotype was expected to be rare and was therefore treated as descriptive.

## 3. Results

### 3.1. Demographic Characteristics

The study sample included 500 patients (250 males and 250 females). Ages ranged from 19 to 71 years (overall mean 40.8 ± 10.0 years). Males ranged from 19 to 71 years (mean 41.3 ± 10.2), and females from 22 to 61 years (mean 40.3 ± 9.7) (Table 1).

### 3.2. Root Morphology, C-Shaped Prevalence, and Pulp Chamber Dimensions

Complete bilateral Vertucci coding was achieved in 494 of 500 patients (98.8%), whereas the remaining 6 of 500 patients (1.2%) exhibited C-shaped canal morphology and were analyzed separately (Table 2).

C-shaped canal morphology was observed in six patients. At the patient level, laterality was right-only in 3 of 6 cases (50.0%), bilateral in 2 of 6 cases (33.3%), and not available in 1 of 6 cases (16.7%). Fan subtype annotation was available for 5 of 6 patients. Because two patients presented with bilateral C-shaped anatomy, subtypes were summarized at the tooth level for 7 mandibular second molars (4 from bilaterally affected patients and 3 from unilateral cases (Table 3).

The prevalence of C-shaped canal configuration was 1.2% (6/500; 95% CI 0.44–2.59%), with 0.4% of males (1/250; 95% CI 0.01–2.21%) and 2.0% of females (5/250; 95% CI 0.65–4.61%). There was no evidence of a sex association (Fisher’s exact test, *p* = 0.216) (Table 4).

**Figure 3:**
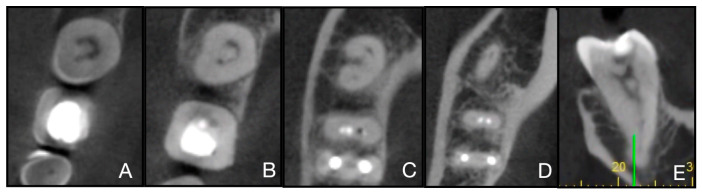
Case 1 of 5 (tooth 4.7, 1st tooth out of 7). (**A**,**B**) Fan subtype C1 is visible in the root’s coronal third; (**C**) Fan subtype C3a is visible in the root’s middle third; (**D**) Fan subtype C4 is visible in the root’s apical third; (**E**) coronal scan of the tooth 4.7 confirms the C-shaped anatomy.

**Figure 4:**
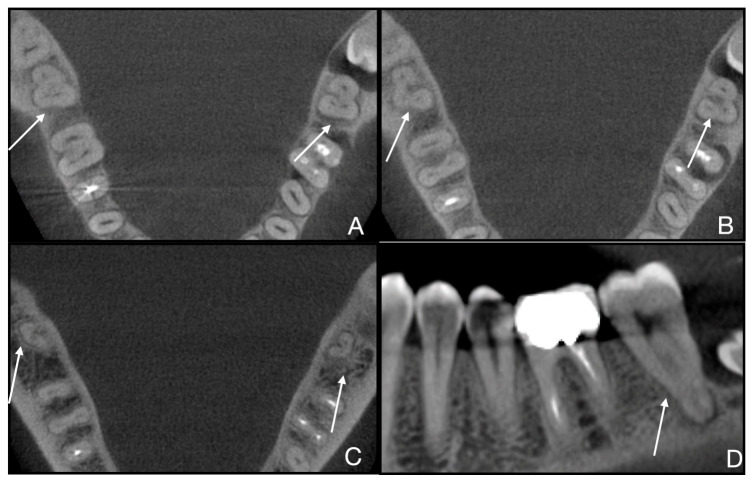
Case 2 of 5 (teeth 3.7 and 4.7, 2nd and 3rd teeth out of 7). (**A**) Fan subtype C3b is visible in the root’s coronal third of teeth 3.7 and 4.7; (**B**) Fan subtype C3b is visible in the root’s middle third of teeth 3.7 and 4.7; (**C**) Fan subtype C3a is visible in the apical third for tooth 3.7 and subtype C4 in the apical third for tooth 4.7; (**D**) sagittal scan of tooth 3.7 shows the typical anatomy of a C-shaped molar.

**Figure 5:**
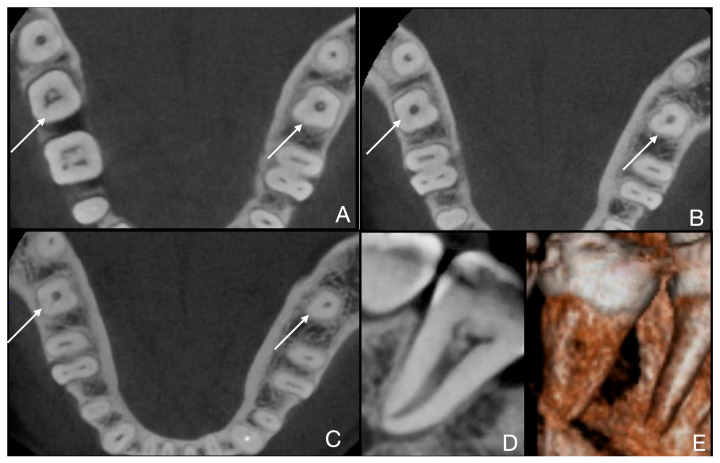
Case 3 of 5 (teeth 3.7 and 4.7, 4th and 5th teeth out of 7). (**A**) Fan subtype C4 is visible in the root’s coronal third for teeth 3.7 and 4.7; (**B**) subtype C4 is visible in the root’s middle third for teeth 3.7 and 4.7; (**C**) subtype C4 is visible in the root’s apical third for teeth 3.7 and 4.7; (**D**) sagittal scan of tooth 4.7 confirms subtype C4); (**E**) the C-shaped anatomy of tooth 4.7 is clearly visible in the 3D reconstruction.

**Figure 6:**
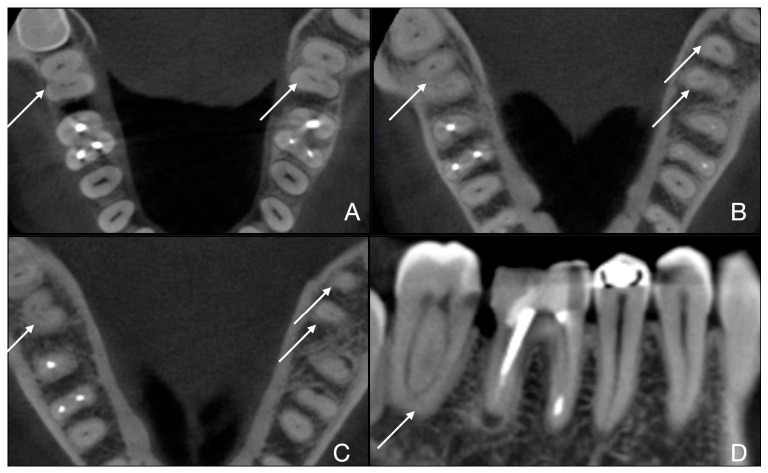
Case 4 of 5 (tooth 4.7, 6th tooth out of 7). (**A**) Fan subtype C3b is visible in the coronal third of tooth 4.7; (**B**) subtype C3b is present in the middle third of tooth 4.7; (**C**) subtype C1 is present in the apical third of tooth 4.7; (**D**) a sagittal scan of tooth 4.7 confirms the C-shaped root anatomy. Conversely, in the same subfigures (**A**–**C**), the tooth 3.7 presents a Vertucci type II configuration in the mesial root and a Vertucci type 1 configuration in the distal root, indicating that bilaterality of C-shaped molars is not present in all cases.

**Figure 7:**
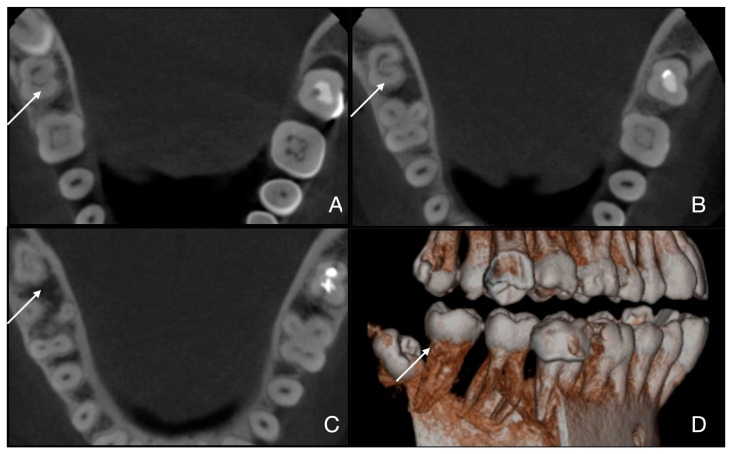
Case 5 of 5 (tooth 4.7, 7th tooth out of 7). (**A**) Fan subtype C1 is visible in the root’s coronal third; (**B**) subtype C1 is also visible in the root’s middle third; (**C**) subtype C1 is visible in the apical root’s third; (**D**) the 3D reconstruction confirms the C-shaped anatomy of the tooth.

However, because only six patients exhibited C-shaped canal morphology, the analysis is underpowered to detect small-to-moderate subgroup differences. Consequently, the observed difference in proportions between males and females should be interpreted as descriptive rather than as robust inferential results. For the same reason (small sample size), it was not possible to establish statistically significant differences in Fan subtype distribution across annotated C-shaped teeth. A tooth-level analysis (*n* = 7 teeth from 5 patients) was conducted, even though more than one subtype was observed in the same root. Consequently, we present the most relevant CBCT images for each case to confirm and describe the C-shaped root canal systems (Figure 3, Figure 4, Figure 5, Figure 6 and Figure 7).

### 3.3. Pulp Chamber Dimensions

The average distance from the pulp chamber roof to the floor was 2.623 ± 0.263 mm on the right and 2.567 ± 0.343 mm on the left, with a statistically significant paired difference (*p* < 0.001). The mean difference between the right and left sides was 0.056 mm (95% CI 0.023–0.089 mm), which is minor in absolute value (Table 5). Between-sex comparisons (Right side *p* = 0.575; Left side *p* = 0.764) did not show statistically significant differences (Table 5).

### 3.4. Canal Configurations in the Mesial and Distal Root (Vertucci Analysis Dataset)

In the predefined Vertucci analysis dataset for mesial roots (*n* = 494; 988 mesial roots), two-canal configurations were most common. Vertucci Types II (52.0%) and IV (26.5%) were the most frequent, followed by Type V (16.5%). Other configurations were rare. In the same dataset, distal roots (988) showed less variability than mesial roots. The most common configuration was Type I at 62.4%, followed by Type III at 29.8%. Table 6 and Table 7 present the distribution of Vertucci canal configurations in mesial and distal canals, while Table 8 illustrates contralateral symmetry, defined as identical Vertucci configurations in contralateral homologous roots (right vs. left) within the dataset (*n* = 494 patients; 988 mesial and 988 distal roots.

### 3.5. Mesial Root Configurations by Sex and Side

Sex-specific comparisons of mesial-root Vertucci configurations showed no statistically significant differences when the right and left sides were analyzed separately (right: χ^2^(6) = 3.72, *p* = 0.715; left: χ^2^(5) = 9.79, *p* = 0.081). Analyses were conducted within the Vertucci dataset, excluding C-shaped cases (males: *n* = 249; females: *n* = 245). On the right side, Type I was observed in 9 of 245 females (3.7%) and 5 of 249 males (2.0%), whereas Type II occurred in 105 of 245 females (42.9%) and 106 of 249 males (42.6%). On the left side, Type I was observed in 6 of 245 females (2.4%) and 2 of 249 males (0.8%), and Type II in 147 of 245 females (60.0%) and 156 of 249 males (62.7%) (Table 9).

## 4. Discussion

This CBCT-based study offers epidemiological data on the root canal anatomy of mandibular second molars from an Italian hospital-based clinical cohort of 500 patients. It employs standardized acquisition parameters and widely used classification systems (Vertucci for root configurations and Fan for C-shaped morphology, when relevant). Since endodontic success relies on accurately identifying, cleaning, and filling the entire canal system, cohort-specific anatomical data remain highly relevant for diagnosis, access planning, and risk minimization, as emphasized in the literature on primary root canal treatment.

At the patient level, conventional anatomy was predominant. Root-based (Vertucci-applicable) analyses were conducted in the predefined Vertucci dataset (*n* = 494/500; 98.8%), which included patients with complete bilateral Vertucci coding of homologous mesial and distal roots, resulting in 988 mesial and 988 distal roots for root-level analyses. The remaining six patients (1.2%) with C-shaped canal anatomy were analyzed separately because a root-based Vertucci scheme does not apply to such systems. This overall pattern aligns with CBCT findings reported in European populations, including Portuguese and Belgian CBCT data cited above [12,13], as well as Greek mandibular first- and second-molar data [22] and Spanish CBCT data on molar root anatomy [23]. In contrast, higher proportions of fused roots and C-shaped morphologies have been reported in Middle Eastern datasets [24]. More recent CBCT cohorts have confirmed that this variability remains substantial across populations. Although direct comparison is limited by differences in case definition and unit of analysis, reported frequencies range from 8.1–8.4% in a recent Saudi cohort [25] to 29.17% in Uyghur adults [26] and 33.1% in a Chinese Kazakh sample [27].

Regarding canal configuration, mesial roots showed greater configurational variability than distal roots, in agreement with previous CBCT studies of mandibular molars. Similar patterns have been reported in other populations. In addition to the Greek data cited above, [22] a Yemeni CBCT study identified Vertucci Type II as the predominant mesial-root configuration [28], whereas an Egyptian cohort showed a predominance of Type IV [29]. Overall, these comparative data indicate that mesial-root anatomy is consistently more heterogeneous than distal-root anatomy, although the dominant mesial subtype may vary across populations. In this dataset, two-canal patterns were most common in mesial roots, with Vertucci Type II (52.0%) and Type IV (26.5%) being the most frequent, followed by Type V (16.5%). Clinically, these configurations raise the risk of missing anatomy and of inadequate shaping, irrigation, and disinfection of intercanal connections—especially when access design or initial exploration relies on assumptions from two-dimensional imaging. In this scenario, selective three-dimensional imaging can help clarify complex internal anatomy when preoperative details are unclear or when intraoperative findings reveal unexpected complexity. In contrast, distal roots showed less variability, with Type I being the most common pattern (62.4%), followed by Type III (29.8%). The same Greek [22], Spanish [23], Yemeni [28], and Egyptian [29] CBCT studies likewise reported a predominance of Type I in distal roots, although the relative frequency of non–Type I patterns varied across cohorts. However, the presence of non–Type I patterns suggests that the distal root should not be assumed to be consistently single-canaled. This has direct clinical implications: even if the distal canal appears simple, thorough exploration and verification are recommended, especially when radiographic clues suggest complexity or tactile feedback indicates bifurcation or merging.

A clinically relevant observation concerns contralateral symmetry. Published CBCT data on mandibular second molars are heterogeneous and not directly comparable because symmetry has usually been assessed at the whole-tooth or root-morphology level rather than by exact agreement of Vertucci type between contralateral homologous mesial and distal roots. Beyond the Greek findings already mentioned [22], Plotino et al. reported 81% perfect bilateral symmetry of root and canal morphology [30], Guo et al. reported 77.1% symmetrical root canal morphology [31], and Yadav et al. reported 98.6% bilateral symmetry of root morphology [32]. These higher values likely reflect broader study endpoints than the root-specific Vertucci agreement used in the present study. By contrast, in the present Vertucci dataset (*n* = 494), where symmetry was defined more strictly as an identical Vertucci configuration in contralateral homologous roots and analyzed separately by root type, symmetry was observed in 27.3% of mesial roots and 59.1% of distal roots. Accordingly, the contralateral tooth may provide supportive information—particularly for the distal root—but it should not be considered predictive of mesial anatomy, where asymmetry was common. Clinically, this supports a cautious strategy: contralateral anatomy can inform expectations, but systematic intraoperative verification remains essential; when uncertainty persists, selective CBCT use may be justified within guideline-based indications. In addition, because mesial-root symmetry was low, any side-specific frequency differences should be interpreted as reflecting inter-individual variability within this cohort rather than as evidence of biologically determined laterality; dedicated paired categorical analyses in larger cohorts would be required to formally test laterality effects.

In summary, the inferential comparisons in this study did not lead to rejection of the null hypothesis of sex-related differences regarding the Vertucci-based hypothesis. No significant sex differences were observed in the Vertucci distributions of the mesial root on either side. In contrast, right–left patterns and contralateral symmetry were examined descriptively and should not be regarded as conclusive evidence for or against laterality effects without specific paired categorical testing.

The prevalence of C-shaped canal configuration in this cohort was low (1.2%, 6/500; 95% CI 0.44–2.59%). Although a higher proportion was observed in females (2.0%, 5/250) compared to males (0.4%, 1/250), there was no evidence of an association with sex (Fisher’s exact test, *p* = 0.216). With respect to the sex-related component of the study’s null hypothesis, these findings did not support rejecting the null hypothesis of no difference in the prevalence of C-shaped morphology in this cohort. However, due to the small number of C-shaped cases, these estimates are inherently imprecise, and the analysis is underpowered to detect small-to-moderate sex effects; therefore, the appropriate conclusion is that there is no evidence of an effect rather than evidence of no effect. Cross-study comparisons also require careful attention to how “C-shaped” morphology is defined and which phenotypes are included under this label because broader thresholds (e.g., transitional or partially fused morphologies) may lead to higher apparent prevalence. This caution is reinforced by recent CBCT series from Nepal [33] and Iran [34]. In this dataset, Fan subtype annotation was available for 5 of 6 patients. Among the annotated teeth (tooth-level; *n* = 7), C1, C3 and C4 patterns were observed. Direct comparisons with the literature should be made cautiously because our data summarize Fan subtypes at the tooth level, whereas many CBCT studies [35,36] report configurations by root third or as longitudinal sequences along the root. These subtype counts are descriptive and should not be interpreted as stable patient-level subtype prevalence, given the small sample size and the one unannotated case.

Methodological considerations are crucial when analyzing CBCT-based anatomical studies. CBCT overcomes major limitations of two-dimensional radiography by providing reliable three-dimensional visualization of canal morphology and root relationships at clinically meaningful resolution. The voxel size used here (0.125 mm) is generally sufficient for assessing main configurations and root relationships under standardized acquisition conditions. In this dataset, the pulp chamber roof-to-floor distance showed a statistically significant right–left difference (right: 2.623 ± 0.263 mm; left: 2.567 ± 0.343 mm; paired *p* < 0.001), but the actual difference was small (mean difference 0.056 mm; 95% CI 0.023–0.089 mm). Its clinical importance should be interpreted with caution, especially given CBCT spatial resolution. Additionally, CBCT cannot resolve microanatomical details visible with micro-CT; therefore, accessory canals and fine apical structures may be underestimated, and prevalence estimates should be considered within these inherent limitations.

Limitations of this study include its retrospective design and potential selection bias, as CBCT scans were obtained for clinical reasons (surgical, periodontal, and endodontic) rather than for population screening, which may affect external validity. Additionally, Fan subtype annotation was unavailable for one C-shaped patient, and laterality information was missing for another, limiting the ability to infer subtype and side-specific details of C-shaped morphology. Coding reproducibility was supported by examiner calibration, independent assessments, and a structured consensus process for cases with discrepancies. Finally, using a predefined rule to assign a single Vertucci type when morphology changes along the root length increases internal consistency but may reduce comparability with studies using different assignment methods; this should be considered when interpreting differences between studies.

Strengths of this study include a balanced sex distribution, standardized CBCT acquisition parameters, and separate analyses of C-shaped phenotypes and Vertucci-based configurations, which avoided mixed denominators and classification inconsistencies. Overall, these findings provide clinically relevant evidence for endodontic diagnosis and treatment planning in an Italian setting by showing greater configurational variability in mesial than in distal roots, indicating that contralateral anatomy may serve as a supportive reference mainly for distal roots and only cautiously for mesial roots, and highlighting that C-shaped morphology, although uncommon, remains clinically relevant when present.

## 5. Conclusions

In this Italian CBCT cohort, mandibular second molars showed low prevalence of C-shaped morphology and greater anatomical variability in mesial than in distal roots. Contralateral anatomy may assist preoperative expectations, especially for distal roots, but should not be considered a reliable predictor of mesial canal configuration. These findings support careful exploration of mesial anatomy and selective CBCT use when conventional assessment does not adequately clarify complex canal morphology.

## Figures and Tables

**Table 1:** Demographic characteristics of the study sample (*n* = 500). Age is presented as mean, SD, and range.

Variable	Total (*n* = 500)	Males (*n* = 250)	Females (*n* = 250)
Age (years), mean ± SD	40.8 ± 10.0	41.3 ± 10.2	40.3 ± 9.7
Age range	19–71	19–71	22–61

**Table 2:** Analysis of denominators (patient-level; *n* = 500).

	*n*	%
Complete bilateral Vertucci coding (Vertucci analysis dataset)	494	98.8
C-shaped canal configuration present (analyzed separately)	6	1.2

**Table 3:** Laterality pattern among C-shaped patients (patient-level; *n* = 6).

Pattern	*n*	%
Right-only	3	50.0
Left-only	0	0.0
Bilateral	2	33.3
Not available	1	16.7

**Table 4:** C-shaped canal configuration (C-SHAPE = YES), stratified by sex. Sex comparison: Fisher’s exact test, *p* = 0.216.

Group	*n*	%
Total (*n* = 500)	6	1.2
Males (*n* = 250)	1	0.4
Females (*n* = 250)	5	2.0

**Table 5:** Pulp chamber roof-to-floor distance (mm) of mandibular second molars (*n* = 500). Values are expressed as mean ± SD.

Group	Right (Mean ± SD)	Left (Mean ± SD)	*p*-Value (Right vs. Left)
Total (*n* = 500)	2.623 ± 0.263	2.567 ± 0.343	0.00081

**Table 6:** Mesial root canal configurations (*n* = 988 mesial roots).

Vertucci Type	*n*	%
I	22	2.2
II	514	52.0
III	16	1.6
IV	262	26.5
V	163	16.5
VI	9	0.9
VII	2	0.2
VIII	0	0.0

**Table 7:** Distal root canal configurations (*n* = 988 distal roots).

Vertucci Type	*n*	%
I	617	62.4
II	41	4.1
III	294	29.8
IV	14	1.4
V	16	1.6
VI	3	0.3
VII	3	0.3
VIII	0	0.0

**Table 8:** Contralateral symmetry of Vertucci configurations (patient-level; *n* = 494).

Root	Symmetry *n* (%)	Asymmetry *n* (%)
Mesial	135 (27.3)	359 (72.7)
Distal	292 (59.1)	202 (40.9)

**Table 9:** Vertucci configurations of mesial roots by sex and side (Vertucci analysis dataset). Values are presented as *n* (%). Sex comparisons were conducted separately by side (right: χ^2^(6) = 3.72, *p* = 0.715; left: χ^2^(5) = 9.79, *p* = 0.081).

Vertucci Type	Right—Males *n* (%) (*n* = 249)	Right—Females *n* (%) (*n* = 245)	Left—Males *n* (%) (*n* = 249)	Left—Females *n* (%) (*n* = 245)
I	5 (2.0)	9 (3.7)	2 (0.8)	6 (2.4)
II	106 (42.6)	105 (42.9)	156 (62.7)	147 (60.0)
III	2 (0.8)	2 (0.8)	9 (3.6)	3 (1.2)
IV	125 (50.2)	118 (48.2)	13 (5.2)	6 (2.4)
V	9 (3.6)	6 (2.4)	68 (27.3)	80 (32.7)
VI	1 (0.4)	4 (1.6)	1 (0.4)	3 (1.2)
VII	1 (0.4)	1 (0.4)	0 (0.0)	0 (0.0)

## Data Availability

The data supporting the findings of this study are not publicly available due to privacy and ethical restrictions related to clinical imaging data.

## Abbreviations

The following abbreviations are used in this manuscript:

CBCT	Cone Beam Computed Tomography
